# From People-Centred to People-Driven Care: Can Integrated Care Achieve its Promise without it?

**DOI:** 10.5334/ijic.7515

**Published:** 2022-11-30

**Authors:** Nicholas Goodwin, Anthony Brown, Hannah Johnson, Robin Miller, K. Viktoria Stein

**Affiliations:** 1 Central Coast Research Institute for Integrated Care, University of Newcastle and the Central Coast Local Health District, Level 10, 77A Holden Street, Gosford, NSW 2250, Australia; 2 Health Consumers NSW, Australia; 3 Reform Office, Strategy Policy and Reform Division, Queensland Health, Australia; 4 Global Engagement for the College of Social Sciences, School of Public Policy, University of Birmingham, UK; 5 Partners Integrating Health and Care, Leiden University Medical Centre, Netherlands

**Keywords:** co-design, community health, coproduction, engagement, empowerment, integrated care, people-centred care

People-centred care seeks to build the skills and resources that individuals and communities need to be articulate and empowered users of health and care services. It is an approach that supports people to make effective decisions about their own health to achieve the outcomes that matter most to them. It enables communities to become active in partnering with care services and contributing to relevant research, education and healthy public policy. Special attention is often given to tackling inequalities by engaging and supporting the voices of marginalised, vulnerable and disengaged people.

As our understanding of integrated care has evolved over the years so the importance of providing a ‘people-centred’ definition to the concept has grown [1]. Still, the application of integrated care in policy and practice has seemingly yet to fully embrace a people-centred approach. This view is fuelled by the observation that integrated care has been primarily used as a programme for other purposes – for example, to reduce acute sector demand and contain costs – and as a consequence of this often fails to deliver on its promise to improve people’s care experiences, achieve better outcomes and address inequalities.

As Charlotte Augst, the former Chief Executive of National Voices, the coalition for health and care charities in England, put it ‘*Integration without personalisation is useless at best, and dangerous at worst. We will only achieve the outcomes we claim to pursue through our integration effort, if we start by asking people and communities what it is that matters to them, and then build a shared, effective, person and community centred response* [2].’

In this editorial, we argue that people-centred care has often remained too passive, condemns patients and carers to subservient roles, and as a result preserves a power imbalance that favours systems and professionals over people and communities. For integrated care to reach its full potential, we instead advocate for a deliberate shift towards ‘people-driven’ care where people have more agency in participating in their health and greater power in decision-making.

Our thinking is derived from the Special Collection on *People-Driven Care: Co-Designing for Health and Wellbeing with Individuals and Communities* that was commissioned to bring together knowledge and evidence of the potential impact of people-driven care, specifically to describe approaches focusing on empowering and engaging people and the role and impact of co-production and co-design. The call for papers yielded 18 papers, of which 14 have to-date been published comprising 8 research papers [3,4,5,6,7,8,9,10], 4 case studies [11,12,13,14], a perspective paper [15], and a research protocol paper [16].

One of the first observations to make from this work is that the state of the evidence on the nature and impact of co-design and co-production in integrated care remains very limited. Whilst our special issue cannot claim to offer more than a sparse reflection on the scope of this evidence, the majority of studies only examined the ‘potential’ importance of pursuing co-design principles and participatory activities in different contexts [e.g. 4,5,6,7,13,14]. Only two research studies provided evidence for more collaborative and people-centred practices resulting in outcomes such as higher satisfaction levels in service delivery and stronger user-professional collaboration [3,8].

However, what is clear from the evidence presented is that the rhetoric supporting people-centred care is not being matched by the reality of delivery. The majority of papers reported either fragmented, or highly passive, involvement of people and communities. Hence, rather than having active roles in co-production and co-design, programmes remained for the most part firmly clinically-led and controlled with little ongoing involvement with the subsequent multi-disciplinary teams and services and an ongoing power imbalance in stakeholder experiences [e.g. 8,9,10,15]. Such observations have been confirmed in other special issues and previous reviews on people-centred care in practice [17,18].

Hidden within the evidence, specifically articulated in case study evidence within the paper by Steele Gray et al. on Goal Oriented Care [12], was that if the person’s needs – as expressed by them – became the core driver of care delivery then this was a much more likely and effective mechanism for ensuring person-centred care coordination at the service and clinical level. The emerging hypothesis, therefore, might suggest that a ‘people-driven’ approach that responds directly to people’s goals and needs is much more likely to result in effective care integration than one that is purposefully designed and led by institutions or health professionals.

If this hypothesis is true then it may explain why some large scale transformation programmes – such as England’s National Integrated Care Pilot Programme [19] – have failed in comparison to those that have a people-driven approach – such as the consumer-owner NUKA model in Alaska [20] and the community-embedded EKSOTE model in Finland [21]. The lack of scientific evidence on this question suggests a significant research gap that needs to be filled. If people-driven care is indeed one of the key ‘signatures’ that determines more or less effective integrated care then this evidence may begin to rebalance and recalibrate how health and care systems should operate.

So what is the likely future prospect for a people-driven approach? The increasing recognition in many health and care systems of the vital role of population health provides some optimism. Empowering and engaging individuals and communities is accepted as a key to health promotion, health equity and better public health. As integrated care systems worldwide seek to become more population-oriented, so co-design and co-production methodologies are likely to grow in response – decision-making becomes more embedded within community settings. It will nonetheless be vital for the success of such approaches to be inclusive and positively respond to diversity and inequalities rather than be dominated by vested interests.

In advocating for the move from people-centred to people-driven care we also recognise that a journal like IJIC must do more to support robust and relevant research in this area. Evidence suggests more attention needs to be paid to co-creation with people within research and to demonstrate inclusive practices [22]. If people-driven care is indeed a more effective route to care integration then research must also tackle its own biases. Power needs to be redistributed from professionals and career academics to embrace a more intimate relationship between research, people and practice [23].
